# Supplementary material for: δ-Catenin Participates in EGF/AKT/p21Waf Signaling and Induces Prostate Cancer Cell Proliferation and Invasion
Source: Int J Mol Sci. 2021 May 18;22(10):5306. doi: 10.3390/ijms22105306 (PMC8157876; doi:10.3390/ijms22105306)
Supplement: Supplementary file 1 [file ijms-22-05306-s001.zip › ijms-1163788-sup-proof done.pdf]

δ-catenin participates in EGF/AKT/p21 signaling and induces prostate cancer cell proliferation and invasion

Yingjie Shen, Hyoung Jae Lee, Rui Zhou, Hangun Kim, Gen Chen, Young-Chang Cho, Kwonseop Kim

This file includes the following subsections:

Supplementary Figure S1-S6

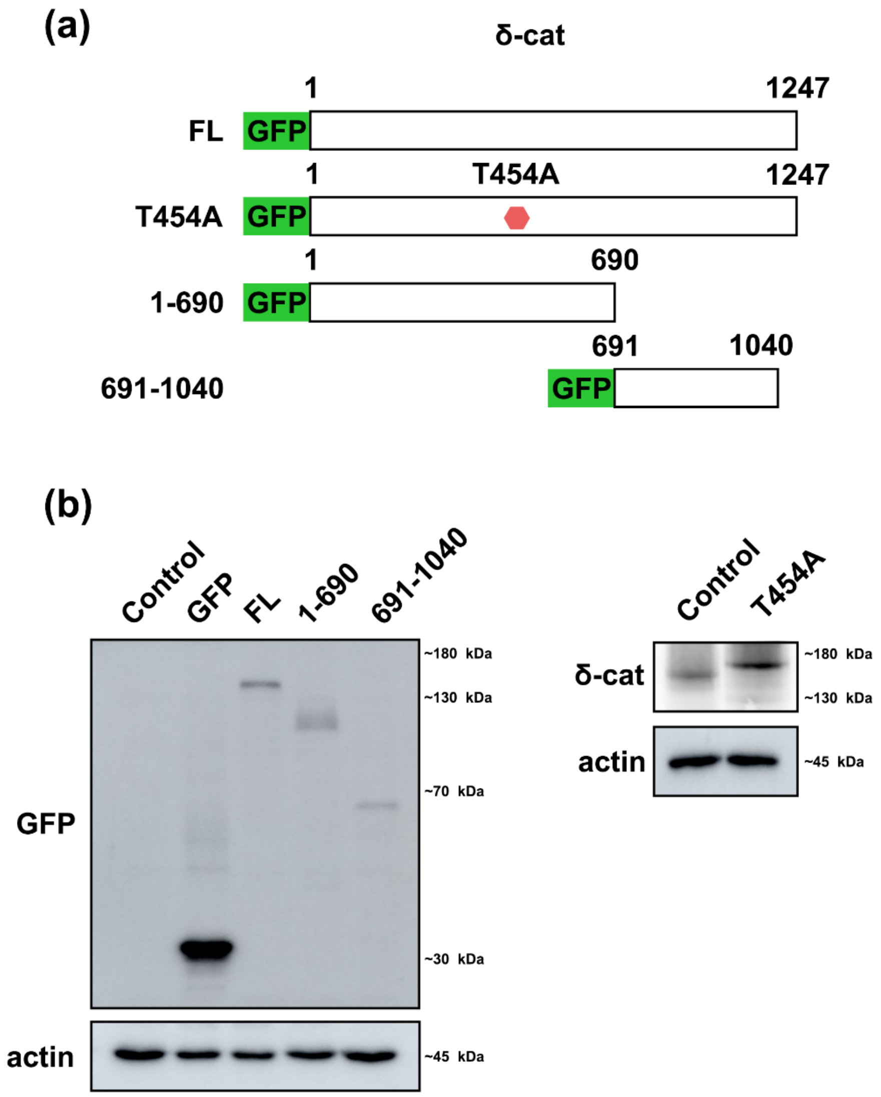

Supplementary Figure S1

(a) GFP plasmid was used as a negative control. GFP plasmid, GFP contacted Full-Length (FL) plasmid or mutant δ-catenin (T454A, 1-690, 691-1040) plasmids were transfected into Rv-1 cells. (b) Cell lysates were immunoblotted with anti-GFP, anti-δ-catenin, and anti-β-actin. This experiment was repeated independently three times.

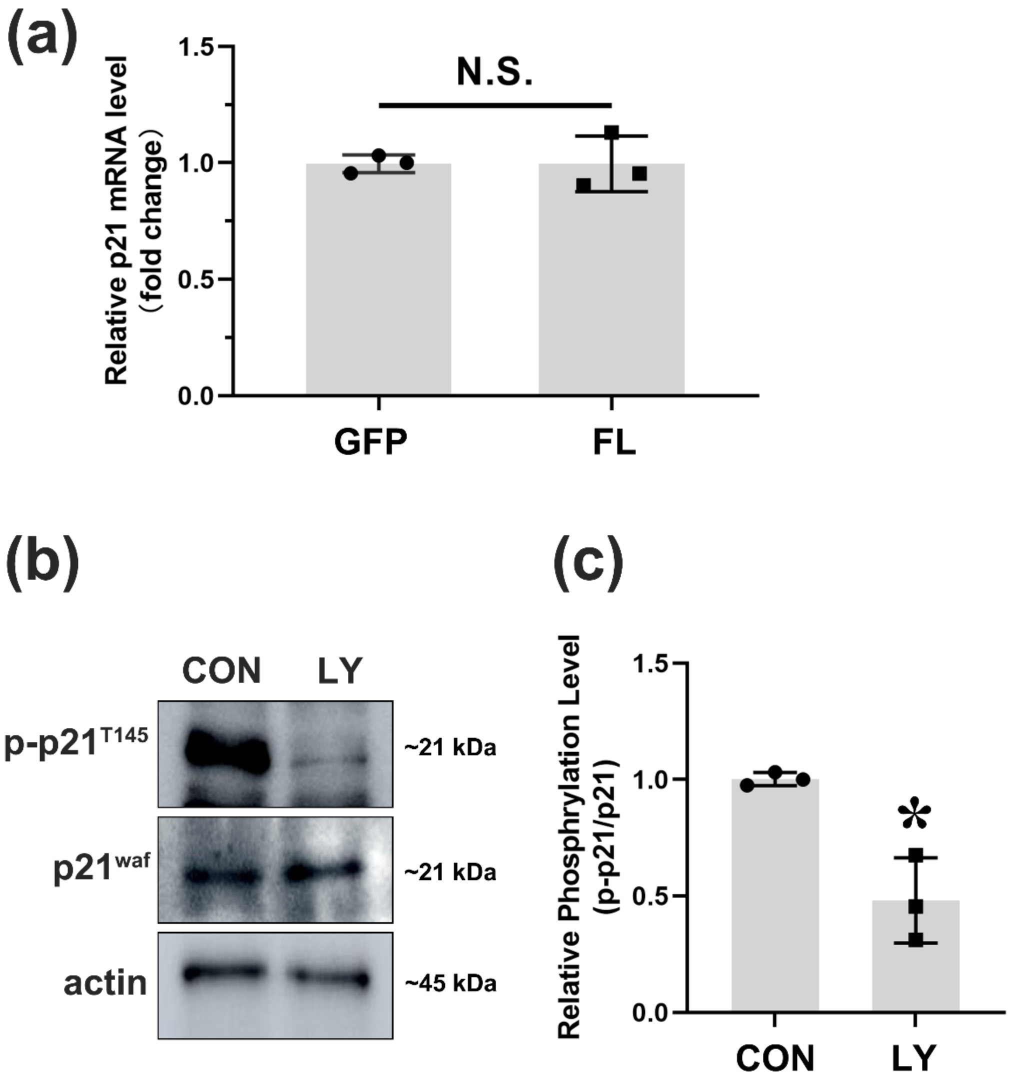

Supplementary Figure S2

(a) Rv-1 cells were transfected with respective plasmid, and the mRNA level of p21 was detected by qRT-PCR, $^{N.S.}$*p > 0.001 vs CON*. (b) RV-1 cells were treated with Ly294002 (1 μM, 24 h). (c) Cell lysates were immunoblotted with anti-p21^{waf}, anti-p-p21^{T145}, and anti-β-actin, **p < 0.001 vs CON*.

All experiments were repeated independently three times, and p values were determined with unpaired Student's t test.

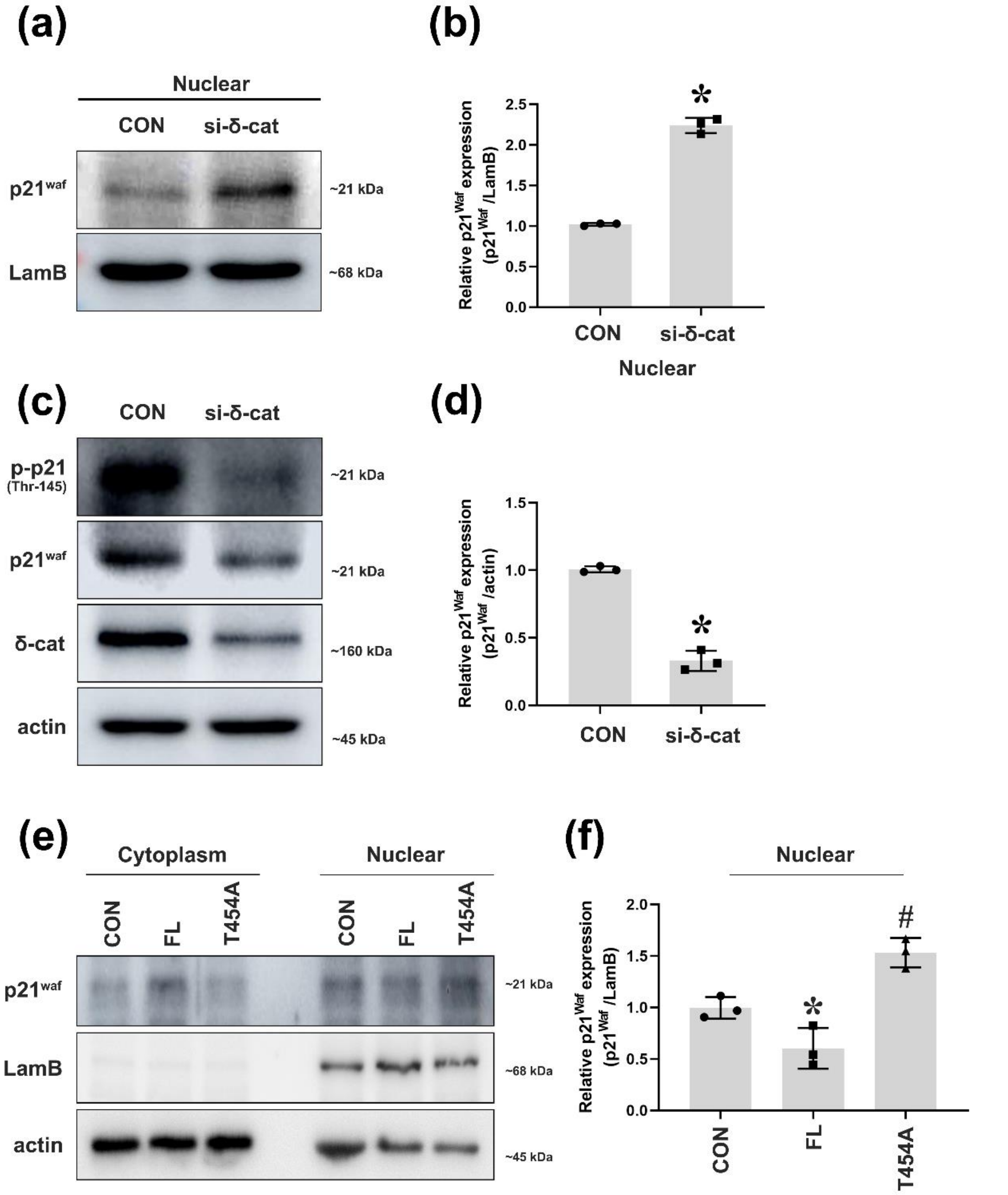

Supplementary Figure S3

(a) RV-1 cells were transfected with si-vector and si-δ-catenin. Nucleus p21 levels were measured by immunoblotted with anti-p21^{waf}, anti-LamB. (b) p21 densitometry data shown in (a) were normalized using LamB (Nucleus) densitometry data, and Quantitate p21/LamB, *$p < 0.001$ vs CON*. (c) RV-1 cells were transfected with si-vector and si-δ-catenin. and cell lysates were immunoblotted with anti-GFP. anti-p-p21^{T145}, anti-p21^{waf}, anti-δ-catenin and anti-β-actin. (d) Quantitation of p-p21/p21 shown in (c), *$p < 0.001$ vs CON*. (e) RV-1 cells were transfected with respective plasmid, and cell lysates were measured by

immunoblotted with anti-p21waf, anti-LamB and anti-β-actin. (f) Quantitation of p21waf/LamB shown in (e), **p* < 0.001 *vs CON*, #*p* < 0.001 *vs FL*.

All experiments were repeated independently three times, and p values were determined with unpaired Student's t test.

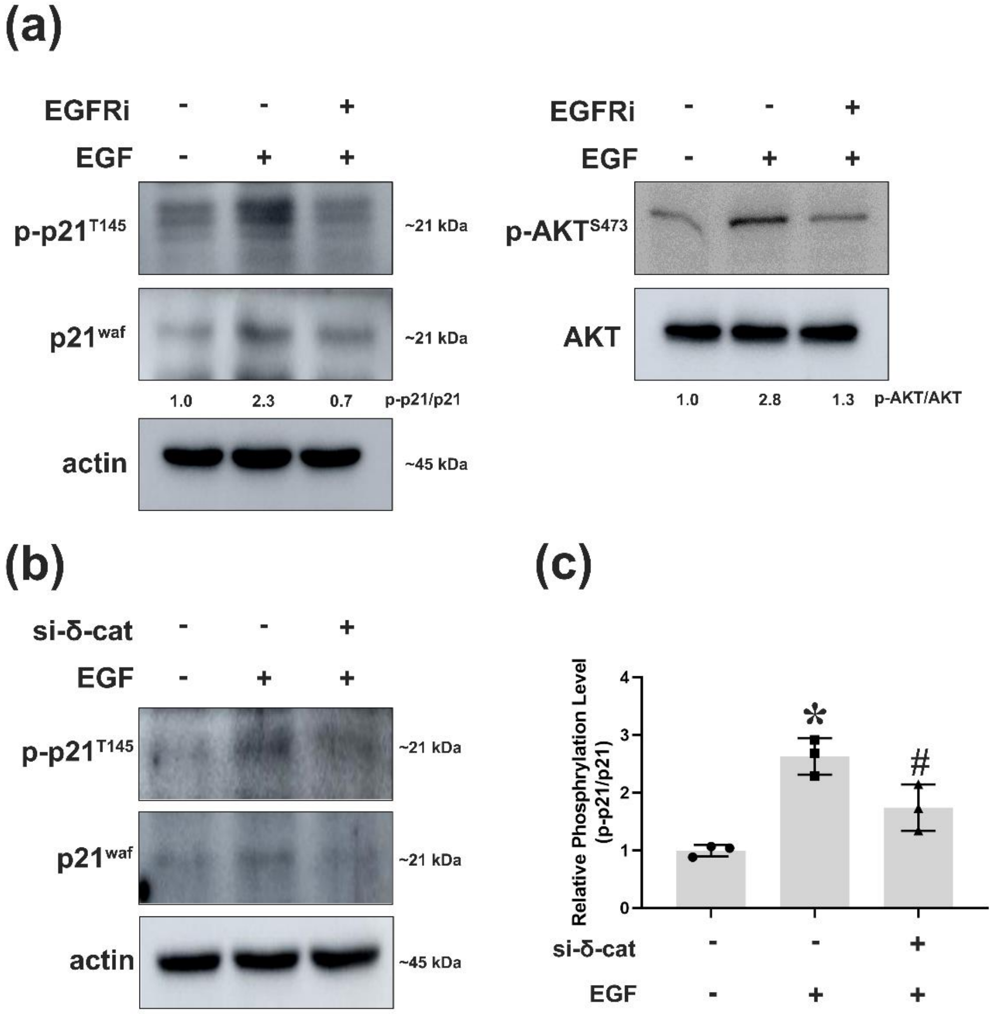

Supplementary Figure S4

(a) Rv-1 cells followed by serum starvation and treatment with gefitinib (10 μM, 2 h) and EGF (100 ng/mL, 5 min), and cell lysates were immunoblotted with anti-p-AKTS473, anti-AKT, anti-p-p21T145, anti-p21waf and anti-β-actin. Quantitation of p-p21/p21 ratio and p-AKT/AKT ratio. (b) Rv-1 cells were transfected with si-vector or si-δ-catenin followed by serum starvation and treatment with EGF. This experiment was repeated independently three times. (c) Quantitation of p-p21/p21 shown in (b). *p < 0.001 vs Control; #p < 0.001 vs EGF.

All p values were determined with unpaired Student's t test.

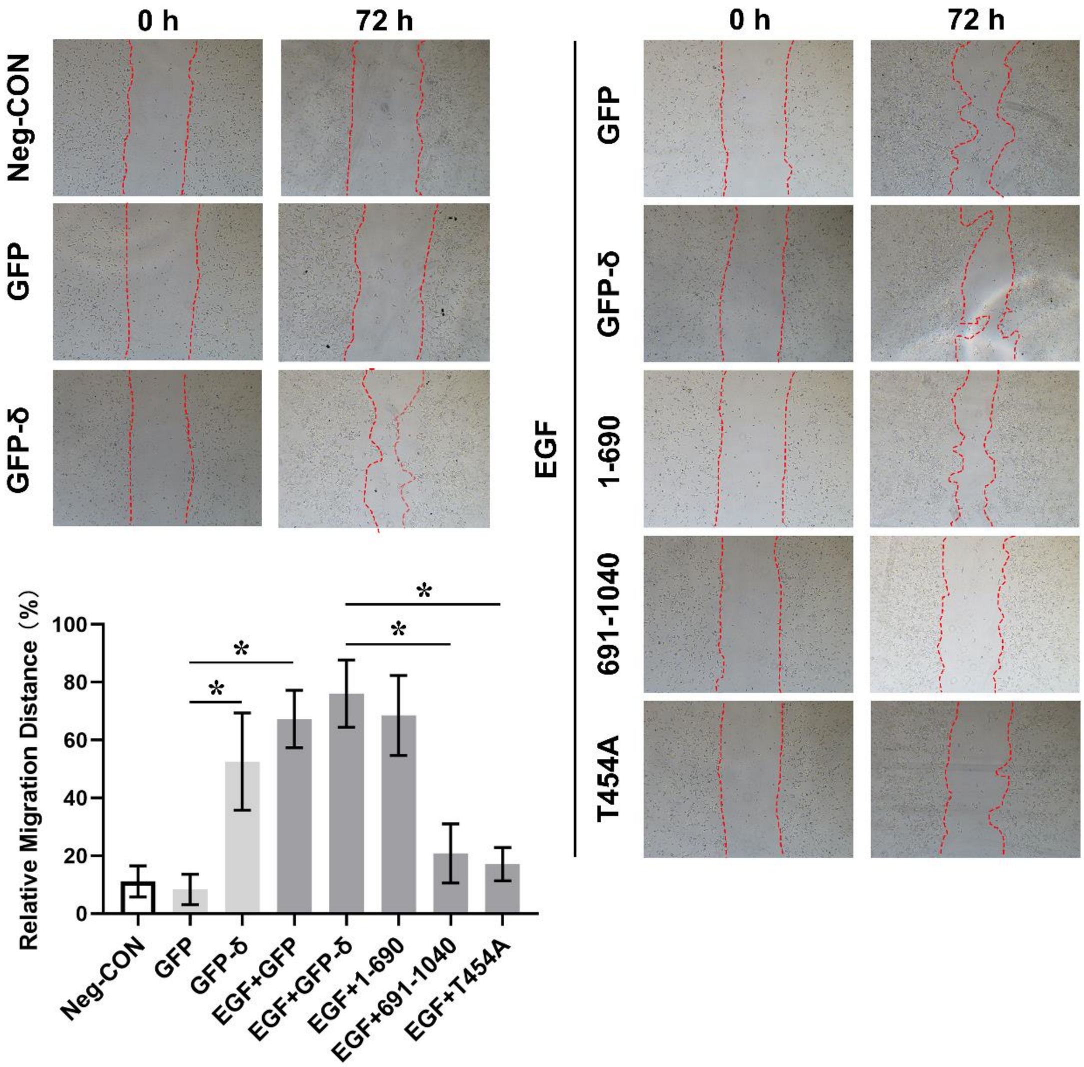

Supplementary Figure S5

RV-1 cells were transfected with respective plasmid, same number of cells were seeded into six-well plate. Scratch wound-healing assay were performed to transfected cells followed low serum medium (0.5% FBS) and treatment with EGF 100 ng/mL. The scratch distance at 0 h and 72 h was detected, and the cell migration rate was counted. This experiment was repeated independently five times. *p values were determined with unpaired Student's t test: *p < 0.001.*

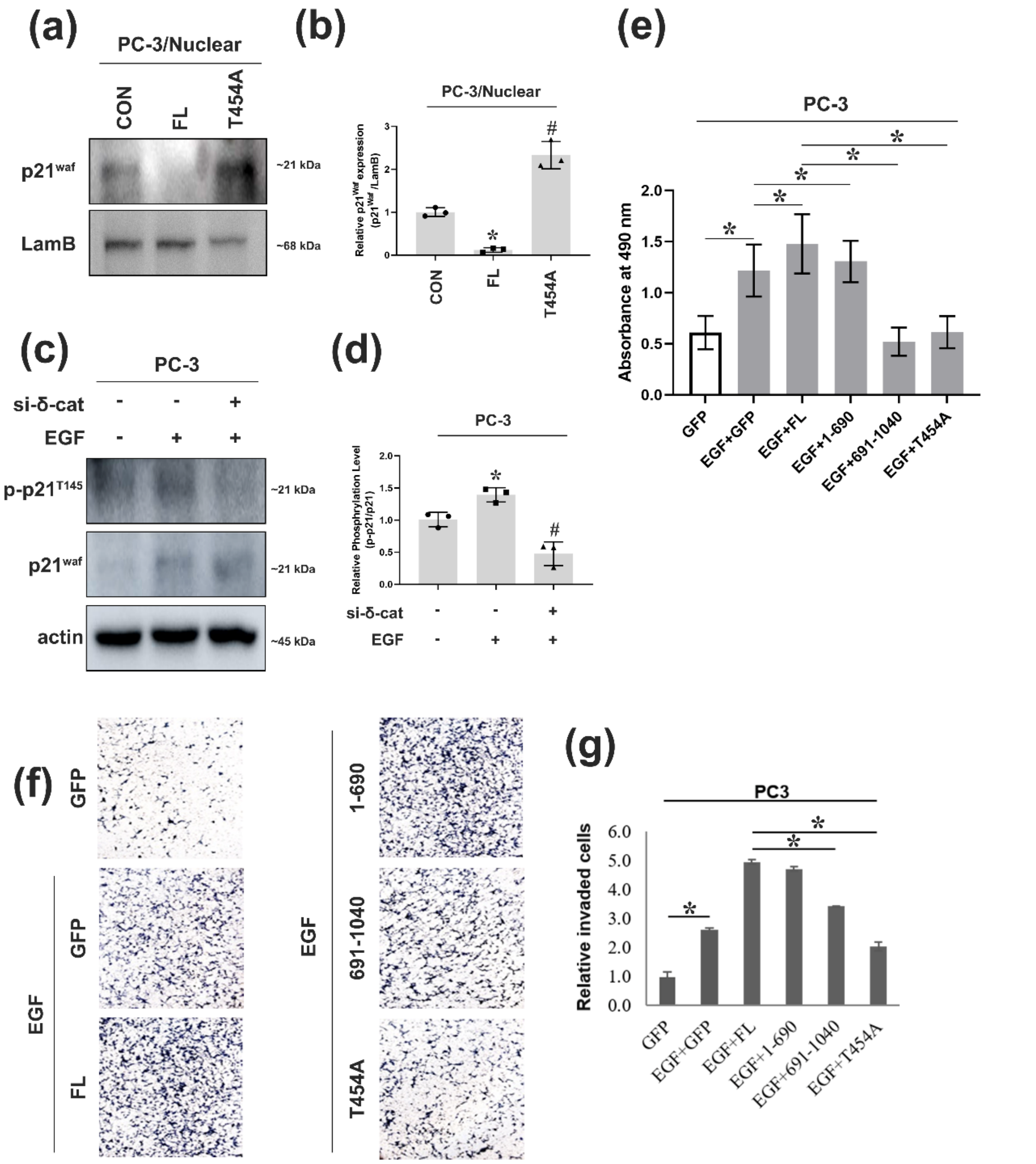

Supplementary Figure S6

(a) PC3 cells were transfected with respective plasmid. Nuclear p21 levels were measured by immunoblotted with anti-p21^{waf}, anti-LamB. (b) Quantitation of p21/LamB shown in (a), **p < 0.001 vs CON, #p < 0.001 vs FL*. (c) PC3 cells were transfected with si-vector or si-δ-catenin followed by serum starvation and treatment with EGF 100 ng/mL for 5 min. (d) Quantitation of p-p21/p21 shown in (c). **p vs Control, #p vs EGF*. (e) PC3 cells were transfected with respective plasmid and then seeded into a 96-well plate. After 12 h, transfected cells were than serum starved followed 12 h treatment with 100 ng/mL EGF or 10% serum. After cells were incubated with CCK8 reagent at 37℃ for 4 h, plates were read at 490 nm to obtain absorbance values. This experiment was repeated independently five times, **p < 0.001*. (f) Transwell

assay was perform with PC3 cells transfected with respective plasmid, treating with EGF 100 ng/mL. (g) Quantitation of positive cells shown in (f), *$p < 0.001$.

Experiments were repeated independently three times, and p values were determined with unpaired Student's t test.
